# Coexistence of Hodgkin's Lymphoma and Tuberculosis in Two Young Adults: Diagnostic and Management Challenges

**DOI:** 10.7759/cureus.85485

**Published:** 2025-06-06

**Authors:** Andika Putra, Mardiah S Hardianti, Noviana Nugrohowati, Afif Rahman, Siswanto S.

**Affiliations:** 1 Department of Internal Medicine, Faculty of Medicine, Public Health and Nursing, Universitas Gadjah Mada/Dr. Sardjito General Hospital, Yogyakarta, IDN; 2 Division of Hematology and Medical Oncology, Department of Internal Medicine, Faculty of Medicine, Public Health and Nursing, Universitas Gadjah Mada/Dr. Sardjito General Hospital, Yogyakarta, IDN; 3 Department of Pathology, Academic Hospital of Universitas Gadjah Mada, Yogyakarta, IDN; 4 Department of Radiology, Faculty of Medicine, Public Health and Nursing, Universitas Gadjah Mada/Dr. Sardjito General Hospital, Yogyakarta, IDN; 5 Department of Physiology, Faculty of Medicine, Public Health and Nursing, Universitas Gadjah Mada/Academic Hospital of Universitas Gadjah Mada, Yogyakarta, IDN

**Keywords:** diagnostic, hodgkin lymphoma, latent tuberculosis, lymphadenopathy, reactivation

## Abstract

Tuberculosis (TB), primarily a pulmonary disease, can affect other organs and has been linked to an increased risk of Hodgkin’s lymphoma (HL). Both conditions share similar clinical manifestations, including fever, night sweats, and weight loss, making diagnosis challenging. We report two cases of HL with a history of TB infection in childhood. The first case involved a 20-year-old female presenting with chronic cough, dyspnea, and weight loss. Imaging revealed an anterior mediastinal mass, and a biopsy confirmed classical HL. The patient received ABVD (doxorubicin, bleomycin, vincristine, and dacarbazine) chemotherapy followed by radiotherapy, leading to partial tumor regression. However, signs of TB reactivation emerged, prompting anti-TB treatment, which alleviated the symptoms. The second case involved an 18-year-old male with a persistent cervical mass initially misdiagnosed as TB lymphadenitis. Despite prolonged anti-TB therapy, the mass persisted and was later diagnosed as HL through immunohistochemistry. He underwent ABVD chemotherapy and radiotherapy, resulting in a favorable response. Together, TB and HL can coexist, complicating diagnosis and management. Clinicians should prioritize thorough diagnostic workups, including histopathology and immunohistochemistry, in patients with persistent lymphadenopathy or atypical TB presentations. Early differentiation between TB and HL is critical to ensure timely and appropriate treatment.

## Introduction

Tuberculosis (TB) and malignancies remain significant global health threats. Approximately 7.5 million people were diagnosed with TB in 2022, with 46% of cases occurring in Southeast Asia [1]. TB is an infectious disease caused by *Mycobacterium tuberculosis*, most commonly affecting the lungs [1]. In pulmonary TB, typical symptoms include a prolonged cough, fever, night sweats, and weight loss. While TB primarily affects the lungs, it can also involve other organs [2]. For example, TB osteomyelitis may appear as a painless bone mass accompanied by constitutional symptoms such as malaise and low-grade fever, often without significant local inflammation [1].

Hodgkin lymphoma (HL) is a monoclonal lymphoid neoplasm that develops in the lymphatic system [3]. HL is a rare malignancy, with an estimated incidence of 2.6 cases per 100,000 people in the United States [3]. Notably, approximately 10% of cancer patients may have active TB [4]. HL typically presents with lymphadenopathy, particularly in the cervical, supraclavicular, and mediastinal regions, along with systemic symptoms including fever, night sweats, and unexplained weight loss. Studies have reported a significantly increased risk of HL in individuals with a history of TB. TB patients show reduced Th1 response and/or enhanced Th2 response, and the severity of the disease is closely related to Th1 response, as the lower the Th1 response, the more severe the disease [5]. Th1/Th2 imbalance leads to impaired immune function and augmented escape of tumor cells, thus promoting tumor formation, which may explain the association between TB and lymphoma. Patients with lymphoma are at an increased risk of developing TB due to their immunocompromised state. The presence of TB can also complicate the clinical course of lymphoma, leading to diagnostic uncertainty and potential delays in treatment. Diagnosing these conditions can be challenging due to their overlapping clinical presentations [4]. Lymphoma and tuberculosis may exhibit similar symptoms, including fever, night sweats, and weight loss. Repeated investigations, especially biopsy and histopathological examination, may help establish a correct diagnosis, as time is of the essence and impacts the prognosis of malignancy. This case report aims to highlight the diagnostic challenges in distinguishing TB from HL due to their overlapping clinical features and to emphasize the increased risk of TB infection in patients with malignancies, particularly lymphoma. We present two cases of HL and TB in young adults with a history of TB infection in childhood.

## Case presentation

Case 1

**Figure 1:**
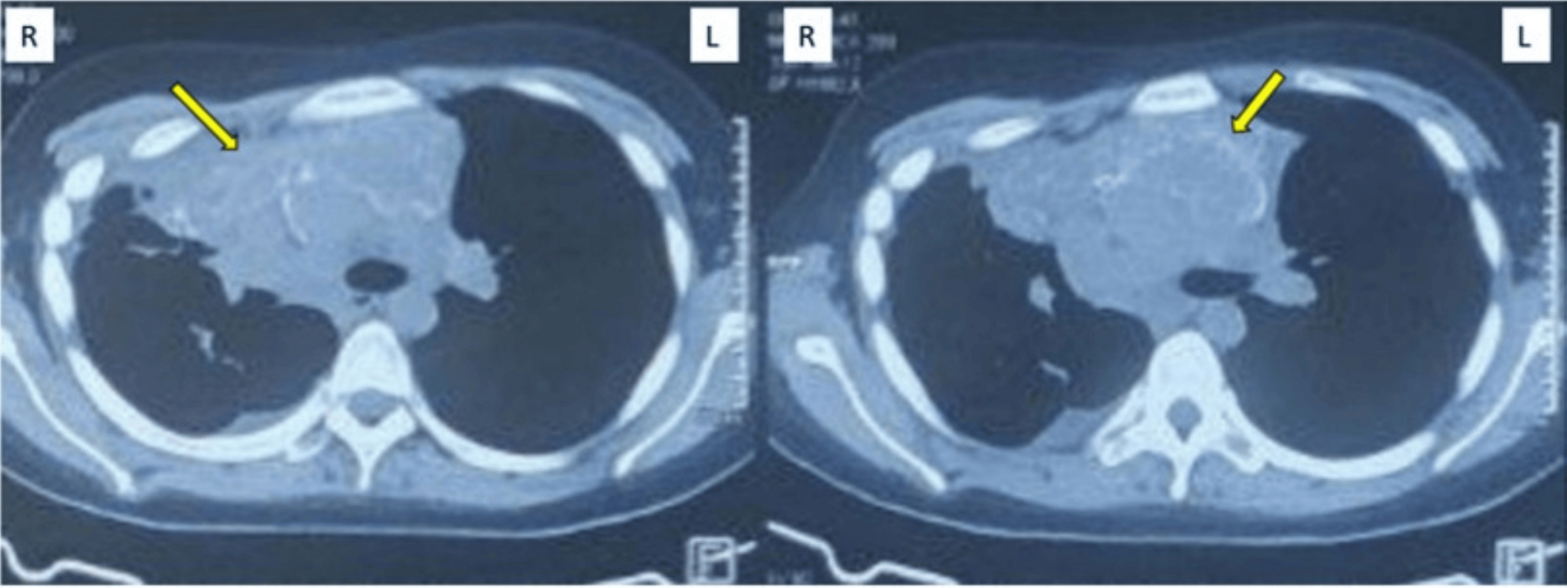
Chest CT revealed a mass in the anterior mediastinum (yellow arrow)

In November 2022, a 20-year-old female presented with a chronic cough, shortness of breath, and weight loss that had persisted for six months. After multiple visits to healthcare facilities and worsening symptoms, she eventually underwent a chest CT scan, which revealed an anterior mediastinal mass (Figure 1).

**Figure 2:**
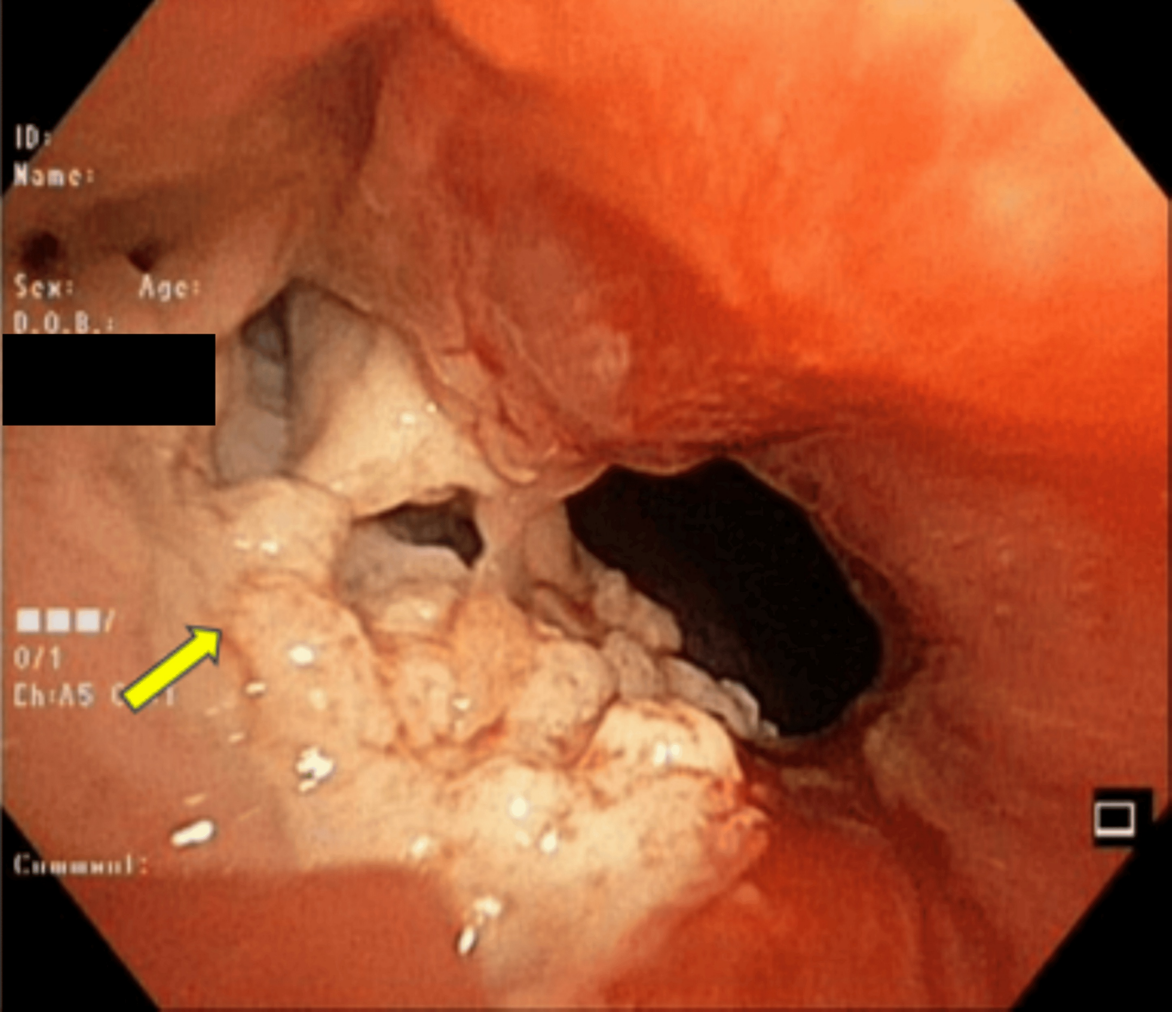
Bronchoscopy showed a visible lesion (yellow arrow) with a narrowed orifice and hyperemic mucosal layer

Bronchoscopy revealed a mass with a friable mucosal layer in the right upper lobe of the lung (Figure 2), which was subsequently biopsied.

**Figure 3:**
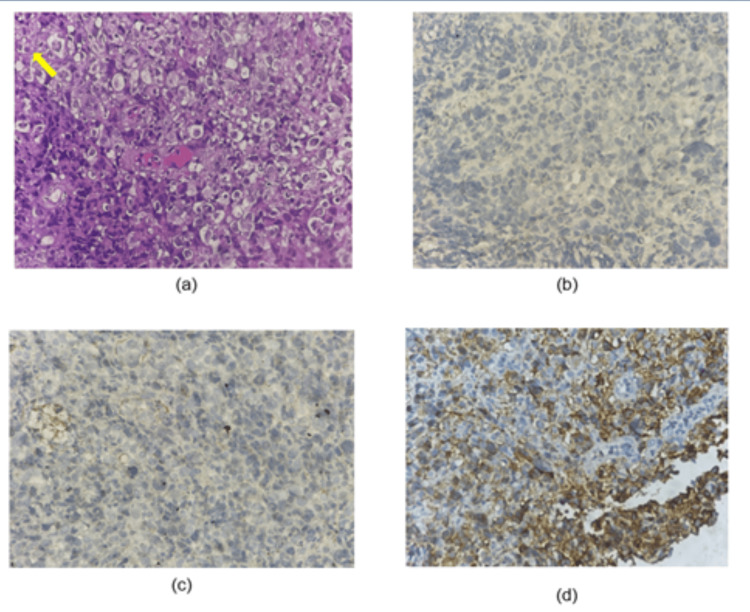
(a) Histopathological findings showing a Reed-Sternberg cell with an "owl-eye" appearance (yellow arrow). Immunohistochemistry examination showing (b) negative LCA, (c) negative CK, and (d) positive CD30. Magnification: 400x LCA: Leukocyte common antigen; CK: Cytokeratin.

Immunohistochemistry (IHC) confirmed a diagnosis of classical HL, with findings of negative leukocyte common antigen (LCA), negative cytokeratin (CK), negative Pax5, and positive CD30 staining (Figure 3). Her Eastern Cooperative Oncology Group (ECOG) performance scale was three. Despite this, she was initiated on ABVD chemotherapy (doxorubicin 25 mg/m², bleomycin 10 units/m^2^, vinblastine 6 mg/m², and dacarbazine 375 mg/m²) on day 1 and day 15, planned for four cycles.

**Figure 4:**
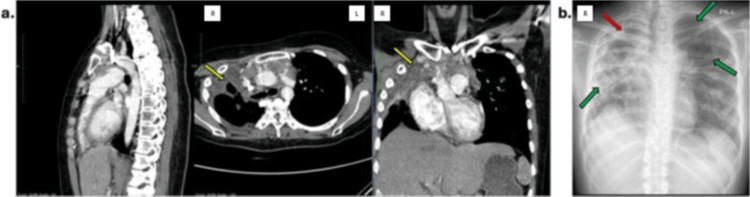
(a) Post-chemotherapy chest MSCT and (b) chest x-ray images revealed a 1.25 × 6.20 × 1.60-cm isodense, fine margin, and irregular lesion in the anterior mediastinum, consistent with a partial response of the mediastinal mass (yellow arrow); reactivated tuberculosis infection, evidenced by a cavitary lesion in the apex of the right lung (red arrow); and multiple hypodense, well-defined, thin-walled, air-filled lesions with regular margins observed bilaterally in the lungs, consistent with pulmonary bullae (green arrows). MSCT: Multislice computed tomography.

After two cycles, her symptoms improved, and she could perform daily activities with no limitations. She completed all four cycles of ABVD, followed by 28 sessions of mediastinal radiotherapy. Treatment-related adverse effects included fatigue, grade two mucositis, and grade one nausea and vomiting; however, these did not lead to treatment interruption or dose modification. Treatment evaluation showed a 55% reduction in tumor size, classified as a partial response according to the Lugano criteria. However, imaging findings suggested TB reactivation, accompanied by recurrent coughing (Figure 4).

Subsequently, two PCR tests, an acid-fast bacilli (AFB) test, and an interferon-gamma release assay (IGRA) were performed, but TB testing was inconclusive. Although overall clinical condition improved, with her ECOG status improving from three to zero and body weight increasing from 35 to 50 kg, she continued to experience occasional coughing. This made it difficult for her to lie flat for the required 45 minutes, thereby delaying the planned PET scan. Despite negative findings across all investigations, the pulmonologist initiated anti-TB therapy based on clinical judgment and supportive imaging evidence. The anti-TB treatment resulted in significant clinical improvements with reduced occasional cough; consequently, the anti-TB regimen was extended to a 12-month duration. In the last follow-up in April 2025 (30 months after the diagnosis), she demonstrated overall wellness, with no restrictions in daily life activities. A PET scan was scheduled for her next visit.

Case 2

**Figure 5:**
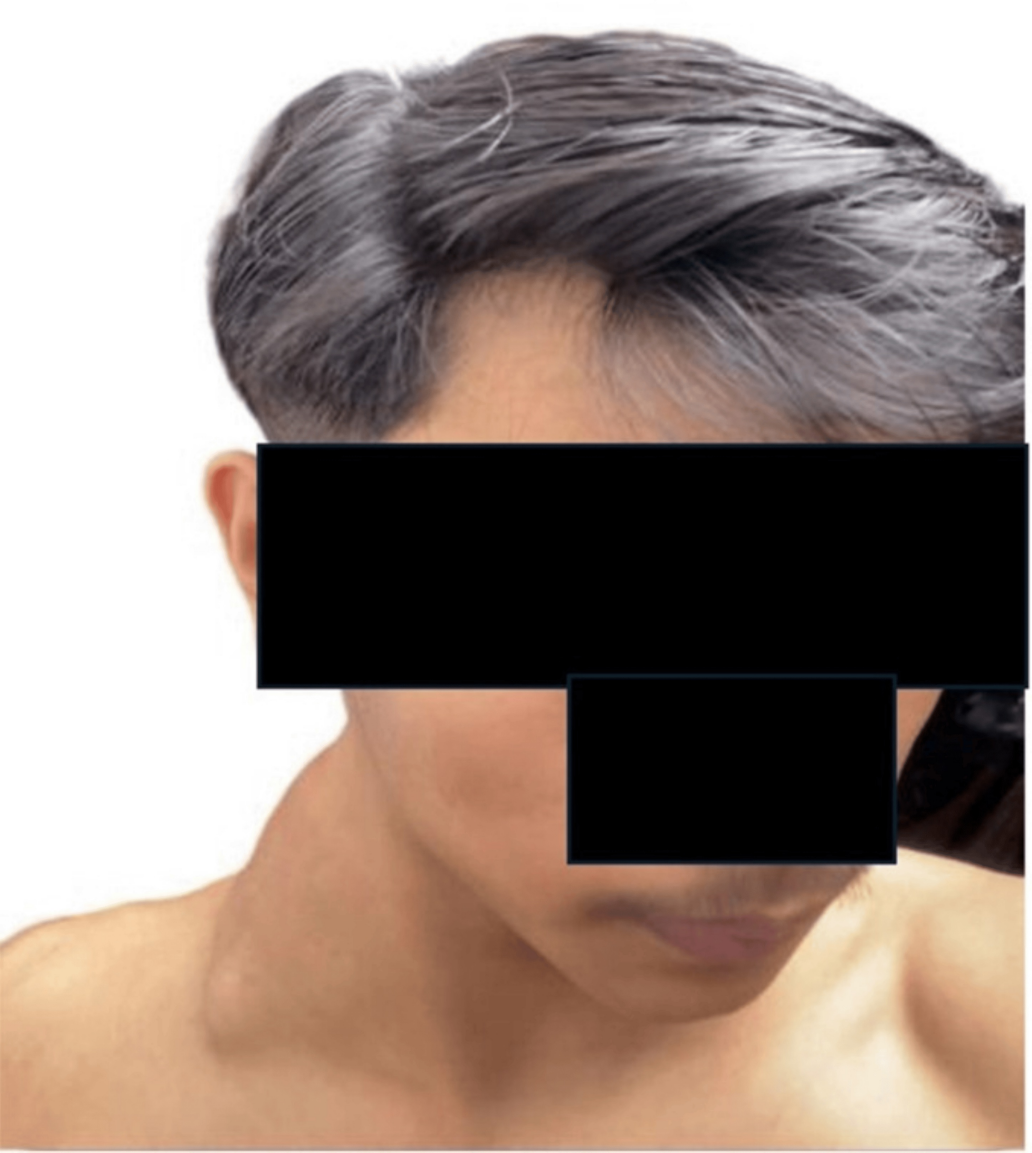
5 x 6 cm mass of the right neck

In June 2023, an 18-year-old male presented with painless, slow-growing masses on the right side of his neck, measuring 5 x 6 cm in diameter (Figure 5). The remainder of the physical examination was unremarkable. Initially, he was diagnosed with lymphadenitis by the general practitioner and treated with antibiotics for one week, but the mass did not reduce in size.

**Figure 6:**
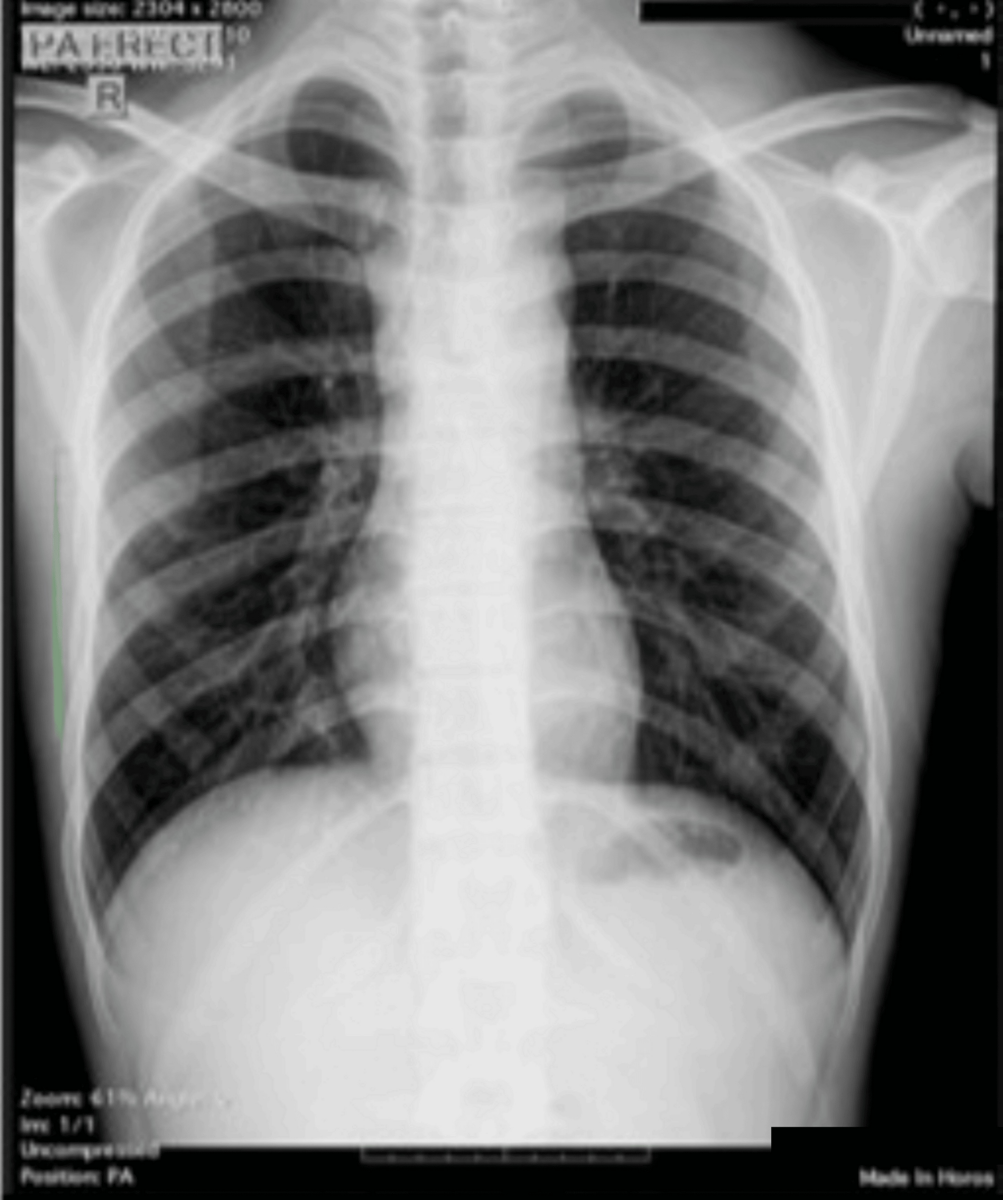
Chest x-ray before treatment showed no abnormality

He was then referred to a pulmonologist, where a chest x-ray appeared normal (Figure 6). A fine needle aspiration biopsy revealed a pattern consistent with lymphadenitis TB. He underwent nine months of treatment with anti-TB medication, but the mass persisted and continued to grow.

**Figure 7:**
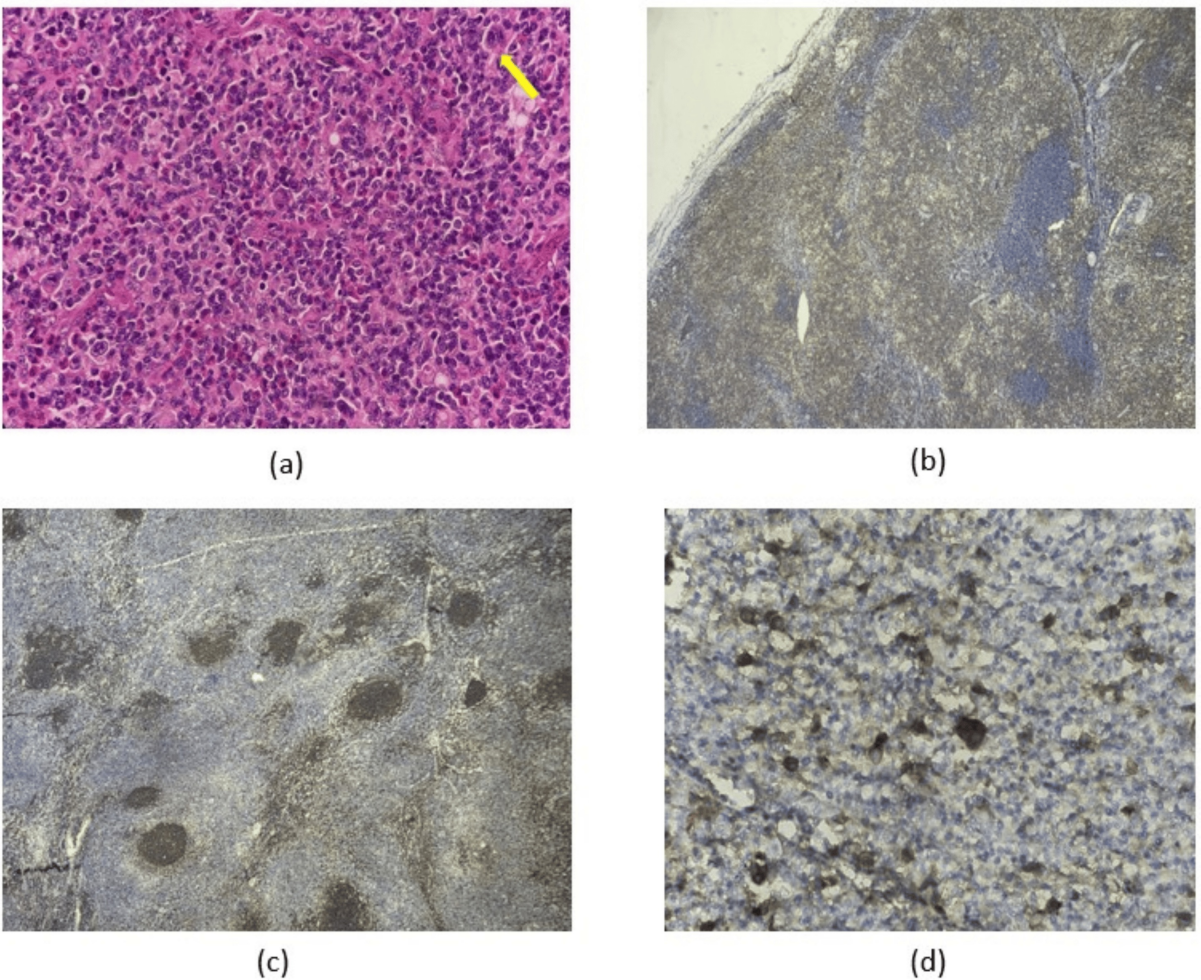
(a) Histopathological findings show a Reed-Sternberg cell with an "owl-eye" appearance (yellow arrow) (400x). Immunohistochemistry examination showing (b) negative CD3 (40x), (c) negative CD20 (40x), and (d) positive CD30 (400x).

He was subsequently referred to a surgical oncologist for an excisional biopsy of the enlarged right cervical lymph node. IHC examination showed positive CD30 staining with Reed-Sternberg cells and was negative for CD20, CD3, and LCA, confirming a diagnosis of HL (Figure 7). The patient was subsequently referred to our department, maintaining a good clinical performance status with ECOG zero. Vital signs and physical examination findings were within normal limits. ABVD chemotherapy, comprising doxorubicin (25 mg/m²), bleomycin (10 units/m²), vinblastine (6 mg/m²), and dacarbazine (375 mg/m²), was administered on days 1 and 15. He completed four cycles of ABVD followed by 32 sessions of radiotherapy. The neck lesion showed partial regression after two cycles of chemotherapy and complete resolution after four cycles.

**Figure 8:**
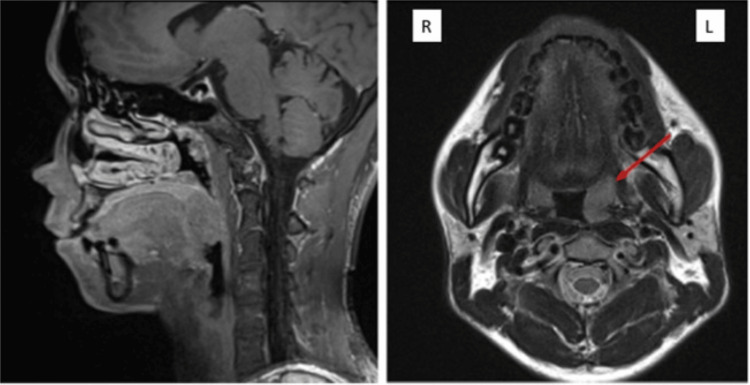
MRI assessment following treatment revealed complete resolution of the cervical lymph nodes and bilateral enlarged palatine tonsil lymph nodes, measuring AP 1.72 x LL 1.77 x CC 2.35 cm (red arrow) AP: Anteroposterior; LL: Laterolateral; CC: Craniocaudal.

MRI imaging demonstrated complete resolution of the cervical lymphadenopathies, although an enlarged palatine tonsil lymph node remained detected and stable at the most recent evaluation in March 2025 (Figure 8). The patient maintained a good performance status throughout the observation period, with no documented weight loss.

## Discussion

These case series present two patients with a history of TB infection during childhood. The first patient showed signs and symptoms of TB reactivation after lymphoma treatment and clinically improved after receiving anti-TB therapy. This underscores the importance of TB screening upon malignancy diagnosis; however, no standardized guidelines currently exist. The second patient was treated with anti-TB therapy for several months, which delayed the accurate diagnosis and treatment for the lymphoma. Since timely intervention is crucial in malignancies, delayed diagnosis can have severe consequences, as demonstrated in previous case reports. Although our patient responded well to chemotherapy, this case highlights the necessity of performing an excisional biopsy with pathological examination as soon as anti-TB therapy fails to yield clinical improvement.

Patients with solid tumors and hematologic malignancies have weakened immune systems due to both the disease and chemotherapy. Given the circumstances, it is reasonable to infer that individuals with cancer are at a heightened risk of TB reactivation [6]. Approximately 10% of cancer patients may present with active TB [7]. A large case series previously reported a higher prevalence of TB among patients with HL [8]. The proportions of TB infection in patients with HL and non-HL were significantly higher than those in benign tumor groups [4]. The impaired T-cell-mediated immunity in HL may increase susceptibility to infections, which are further aggravated by HL treatments like chemotherapy, raising the risk of TB reactivation, which may explain the finding in our first case [4]. This finding also aligns with a previous report of TB reactivation showing cavitary lesions, particularly in the upper lobes of the lungs, and the possibility of infiltrates in the apical-posterior segments of the upper lobes [9]. Conversely, chronic inflammation caused by TB can lead to immune dysregulation, creating a microenvironment that may promote lymphomagenesis, suggesting a potential bidirectional link between the two diseases [4].

Immunocompromised individuals frequently exhibit atypical clinical features, making the diagnosis of TB in these patients difficult. Therefore, it is recommended to screen for latent TB infection and evaluate the need for preventive therapy to reduce the risk of TB reactivation [10]. Misdiagnosis or delayed diagnosis of both TB and lymphoma can occur due to overlapping clinical manifestations, for instance, lymphadenopathy, cough, fever, loss of appetite, loss of weight, and night sweats. Immunosuppression remains a key factor in mycobacterial infections among lymphoma patients, with TB being a major cause of mortality in these cases. In a recent report, three patients with a presumed diagnosis of TB on clinical findings (without bacteriological confirmation) were eventually diagnosed with lymphoma [11].

Other proposed pathophysiological mechanisms of tuberculosis-induced malignancy include chronic inflammation that promotes cell proliferation, mutagenesis, and angiogenesis, as well as the inhibition of apoptosis and direct DNA damage by *Mycobacterium tuberculosis*. It has been proposed that a variety of mycobacterial cell wall components can generate reactive oxygen species and nitric oxide, both of which are involved in mutagenesis [12]. Malignant hematological tumors may develop as a result of a compromised immune system induced by chronic inflammation associated with tuberculosis [4]. In a healthy individual, immunity against TB primarily relies on an induced cellular immune response mediated by CD4+ T lymphocytes [13,14]. However, it has been found that HL can inhibit this response, creating a favorable environment for TB infection or reactivation [15].

The tuberculin skin test (TST) has been widely used as a screening method for diagnosing latent tuberculosis infection [16]. However, due to its low specificity and sensitivity, a combined IGRA and TST screening has been suggested for patients at high risk of tuberculosis reactivation, such as immunosuppressed patients [16]. Despite these advancements, biopsy with histopathological examination remains the gold standard for TB diagnosis [12]. TB lesions often present with typical caseating or necrotizing granulomatous lesions, which can also be found in HL. On the other hand, HL is characterized by the presence of Reed-Sternberg cells [3]. However, these hallmark features are not always evident, necessitating further confirmation through IHC. IHC may show positive staining for CD68 and CD3 in TB, while Reed-Sternberg cells typically express CD30 and CD15 in HL. Therefore, immunohistochemistry is essential for accurate assessment [12].

Although TB and malignancy associations are known, diagnostic uncertainty between TB and HL, a common cause of lymphadenopathy in children and young adults, remains a global challenge [17]. The foundation for clinical differentiation between the two entities remains to be histopathological and microbiological analysis [14]. Diagnostic difficulties can lead to delays in the diagnosis and treatment of lymphoma and worsen the prognosis [17].

The limitation of this study is that it presents only two cases, which are inadequate to establish conclusive evidence regarding TB screening in patients with lymphoma. Additionally, our case lacked microbiological confirmation of TB, which would have been beneficial if available. Further research is required to develop a better understanding and address factors affecting the diagnostic delay of lymphoma to improve patient care.

## Conclusions

The interaction between TB and malignancy is bidirectional - TB may promote tumorigenesis, while malignancy can lead to TB reactivation. Further investigation is warranted to evaluate the screening of latent TB in the context of malignancy. Both HL and TB infections share overlapping clinical features, which can lead to diagnostic confusion and delayed treatment. In cases where a patient diagnosed with TB does not respond to standard therapy, alternative diagnoses such as atypical mycobacterial infection, drug-resistant TB, or concomitant malignancy, particularly lymphomas and lung cancer, should be considered. A patient's immune system is impaired due to cancer and treatment-related issues; therefore, it becomes rational to screen for latent tuberculosis as soon as a cancer diagnosis is made.
